# Mobile Health for Smoking Cessation Among Disadvantaged Young Women During and After Pregnancy: User-Centered Design and Usability Study

**DOI:** 10.2196/24112

**Published:** 2021-08-04

**Authors:** Marloes E Derksen, Monique WM Jaspers, Sander van Strijp, Mirjam P Fransen

**Affiliations:** 1 Department of Public and Occupational Health Amsterdam Public Health Research Institute Amsterdam UMC, University of Amsterdam Amsterdam Netherlands; 2 Department of Medical Informatics Amsterdam Public Health Research Institute Amsterdam UMC, University of Amsterdam Amsterdam Netherlands

**Keywords:** think aloud, heuristic evaluation, usability, mHealth, game elements, smoking prevention, user-centered design, mobile phone

## Abstract

**Background:**

Smoking prevalence during and after pregnancy remains high among socioeconomically disadvantaged women. Mobile health (mHealth) apps with game and social support elements seem promising to support smoking cessation.

**Objective:**

This study aims to describe the user-centered design and usability evaluation of Kindle, an mHealth app with game and social support elements, to support disadvantaged young women during and after pregnancy through the first stages of smoking cessation.

**Methods:**

Disadvantaged women (n=9), members of their social networks (n=4), and nurses supporting these women (n=51) were informants throughout the iterative prototype development of Kindle according to the International Organization for Standardization 9241-11:2018. Specific phases included understanding the context of use through secondary analysis of qualitative interview data (phase 1), establishing the user and organizational requirements (phase 2), production of design solutions (phase 3), and usability inspection of the prototype through a heuristic evaluation (3 experts) along with user testing by a think aloud method (5 disadvantaged women and 5 nurses; phase 4). Usability problems were categorized according to the principles of the Healthcare Information and Management Systems Society.

**Results:**

Phase 1 resulted in an understanding of the VoorZorg program and the needs of VoorZorg nurses and clients (eg, focus on early stages of change and building new supportive networks to aid clients in smoking cessation). In phase 2, we established requirements (n=22; eg, mHealth app, secure communication between nurses and clients, easy-to-use interfaces, inclusion of game elements, and tailoring at early stages of change in smoking cessation). Phase 3 resulted in a prototype of Kindle, combining the interface for nurses and clients, including the following functionalities: personal goal setting with earning points; secured chat function between nurses and other clients; and tips, diary, and profile creation. The heuristic evaluation and thinking aloud method in phase 4 revealed 78 usability problems in the interfaces. Most usability problems concerned *simplicity* (eg, unclear clickable button) and *naturalness* (eg, unclear icon).

**Conclusions:**

The user-centered design and usability testing of the mHealth app Kindle yielded useful insights. The involvement of end users, specifically socioeconomically disadvantaged women during and after their pregnancy, resulted in a prototype that met their needs and requirements (eg, mHealth app, secure communication between nurses and clients, easy-to-use interfaces, inclusion of game elements, and tailoring to the early stages of change in smoking cessation) to achieve readiness for smoking cessation. Moreover, the usability evaluation by end users and experts revealed unique usability problems for this population. These insights allow for further optimization of Kindle and encourage future studies to engage disadvantaged populations in all phases of mHealth intervention design and usability testing.

## Introduction

### Background

Tobacco smoking among pregnant women accounts for a substantial proportion of preventable morbidity and mortality [1]. Smoking cessation among pregnant women not only benefits their own health but also reduces the risks of miscarriage, preterm birth, and low birth weight [2]. Moreover, cessation of smoking after pregnancy prevents their offspring from secondhand smoke exposure and, consequently, from diseases linked to secondhand smoke exposure, such as sudden infant death syndrome and respiratory diseases [2]. Strong predictors of smoking prevalence among women in Europe and the United States are low levels of educational attainment, health literacy, and socioeconomic status [3-5].

Mobile health (mHealth) apps appear to have positive effects on smoking cessation [6,7], and the inclusion of multiple game elements seems particularly promising [8]. In general, pregnant women have been found to frequently use eHealth and mHealth [9] and consider mHealth as a useful and playful tool [10]. In particular, the functionalities to interact with other mothers are useful, yet the quality of these web-based communities has been criticized by women. Moreover, women have indicated that interactive functionalities could be enriched by a direct chat with their health care professionals in addition to face-to-face care [10] and that they prefer easy-to-use interfaces [10]. Women have also signaled that they are more likely to be influenced by pregnancy-related information retrieved from an mHealth pregnancy app than widespread internet use [9]. However, women lack mHealth apps that allow personalization and have concerns about data security [10].

Although smoking during pregnancy is a problem among disadvantaged women (ie, those with a lower educational level, unplanned pregnancies, and additional risk factors [3]), the positive effects of mHealth interventions on smoking cessation are not evident in disadvantaged populations, as these interventions show only few improvements in health outcomes in disadvantaged populations [11]. Another review of disadvantaged patients with diabetes showed that mHealth interventions should be improved in terms of access, design, and usability [12]. As mHealth interventions are typically designed with minimal involvement of end users [13], the effectiveness of mHealth interventions among disadvantaged populations might be improved by a user-centered design approach. In a user-centered design approach, users influence how a design takes shape by providing input at subsequent design phases, typically during requirement gathering and usability testing. The added value of a user-centered approach has been demonstrated by a meta-analysis of randomized controlled trials on serious games for health lifestyle promotion. The effectiveness of participatory design depends on roles (eg, informants and co-design) and game design elements (eg, game levels or challenges) [14]. However, to the best of our knowledge, few studies have followed a user-centered design approach in the design of mHealth apps for disadvantaged populations [15,16].

In the Netherlands, the most disadvantaged pregnant women are offered a preventive care program called VoorZorg, which is supplementary to standard maternal care in the Netherlands [17]. The program resembles the nurse-family partnership developed in the United States [18] and the family-nurse partnership implemented in the United Kingdom [19]. These women are supported on multiple domains (eg, personal development and health promotion) [20] by certified, specialized nurses during home visits lasting 2.5 years.

On enrollment in VoorZorg (at 16-28 weeks of gestation), 43% of the women smoked. This reduced to 33% at 32 weeks of gestation and to 48% at 8 weeks after delivery [20]. VoorZorg nurses find it hard to support these women in smoking cessation due in large part to the use of support methods that do not fit the needs of women. Moreover, women are poorly motivated to stop smoking because of multiple stressors and other challenges they face [21]. Disadvantaged women appear to be in the early stages of change for smoking cessation, and their social networks mainly play a negative role in their smoking cessation efforts [22]. These insights reveal a misalignment between these women’s contexts and traditional action-oriented interventions for smoking cessation. Without planned interventions, these women will remain stuck in the early stages [23]. VoorZorg nurses and their clients are thus in need of an innovative intervention to move women through early stages of change with supportive social networks to stimulate them to quit smoking. As such an intervention is still nonexistent and recognizing the use of mHealth apps seems promising, we developed and evaluated a smartphone prototype with game elements to support disadvantaged young women during and after pregnancy with smoking cessation named *Kindle*.

### Objectives

This paper aims to report the user-centered design process and usability evaluation of Kindle by disadvantaged women and health care professionals and provides insights and recommendations regarding the design of mHealth apps for disadvantaged user populations.

## Methods

### Design

In this user-centered design study, we included women from the VoorZorg program, members of their social networks, and VoorZorg nurses as informants in the design of Kindle. The study was conducted from June 2017 to May 2018 in the Netherlands. The design team members were designers affiliated with Waag (an organization developing innovative, inclusive technology for society), researchers (MD, PhD student Public Health; MF, PhD Public Health; SS, Master of Science student Medical Informatics; and MJ, professor Medical Informatics and human factors engineering expert), and a VoorZorg program representative of the Netherlands Centre for Preventive Youth Health. Kindle was inspected for usability by 3 human factors engineering experts under the supervision of MJ and tested by representative end users in June 2018.

Informed consent was obtained from all participants in this study. The Medical Ethics Review Committee of Amsterdam University Medical Centers confirmed that the Medical Research Involving Human Subjects Act does not apply to this study, and therefore, no official approval of the committee was required. We used the Statement on Reporting of Evaluation Studies in the Health Informatics framework to report our study [24].

In the iterative user-centered design process of Kindle, we followed the standards of the International Organization for Standardization 9241-11:2018, which supports the identification and planning of effective human-centered design activities. These design activities entail four phases: (1) understanding and specifying the context of use, (2) specifying the user and organizational requirements, (3) production of design solutions, and (4) evaluating design solutions [25].

The usability of the Kindle was assessed as part of the fourth phase. Usability is generally defined as “the extent to which a system, product, or service can be used by specified users to achieve specified goals with effectiveness, efficiency, and satisfaction in a specified context of use” [25]. As no usability method is effective in all circumstances, a combination of usability methods that complement each other is generally preferred [26]. One expert-based usability inspection method is heuristic evaluation. During heuristic evaluation, a small number of human factors engineering experts evaluate a user interface according to a set of heuristics, which likely results in high-quality results over short periods [26,27]. The Healthcare Information and Management Systems Society (HIMSS) principles are commonly used to categorize usability problems. These principles include simplicity, naturalness, consistency, minimizing cognitive load, efficient interactions, forgiveness and feedback, effective use of language, effective information presentation, and preservation of context [28]. The think aloud method is a low-threshold, user-based usability testing method [29]. Think aloud entails end users performing tasks with the user interface while verbalizing what they are doing and provides insight into the causes of usability problems encountered by end users, thereby providing suggestions for redesign [26].

### Phase 1: Understand and Specify the User Context

We performed a secondary analysis on unpublished, empirical data from our earlier qualitative studies among VoorZorg nurses [21], clients, and the social networks of clients [22]. MD inductively coded text fragments describing the user context, which were then discussed with MF and MJ.

### Phase 2: Specify User and Organizational Requirements

#### Overview

For the specification of user and organizational requirements, we used the same secondary analysis of our qualitative interviews in phase 1. Moreover, we held two intervention design inquiry sessions, one among VoorZorg nurses and one among clients and members of their social networks.

#### Participant Recruitment

Women who participated in VoorZorg, members of their social networks, and VoorZorg nurses were involved as informants in the user-centered design process of Kindle (ie, phases 2, 3, and 4). Participant recruitment started by informing managers of Youth Health Care Organizations executing the VoorZorg program and asking their permission to contact their nurses. The nurses were then informed about the study during a conference. Subsequently, nurses were asked via email whether they were willing to participate and willing to invite their clients for this study. In general, the target population of VoorZorg consists of women who at enrollment are (1) up to 28 weeks of gestation of their first (live born) child, (2) aged <26 years, (3) lower educated, (4) proficient in Dutch, and (5) have minimally one additional risk factor (eg, alcohol or drug use, financial difficulties, domestic violence, and psychosocial symptoms). Less than 1% of the births per Dutch municipality qualify to enroll in VoorZorg [17]. Most of these women (98%) had four or more risk factors [30]. Next, all participating clients were asked, by the researcher (MD), to invite members of their social networks to design sessions.

Inclusion was based on consecutive sampling. Women (ie, clients) were included when they were registered in the VoorZorg program and were in any of the stages of change in smoking cessation [23]. Members of the social networks of these clients were included when they were related to clients meeting the inclusion criteria. For nurses to be included, they must have worked as a VoorZorg nurse in the Netherlands for a minimum of 6 months.

#### Response and Characteristics

The mean age of the clients (n=9) was 24 years (SD 4.29). Most clients had a child aged <1 year (n=6) or had a child aged >1 year (n=3) and were in the early stages of change in smoking cessation [23], smoking 2.5-20 cigarettes per day. Most pregnancies were unplanned, with one client being pregnant during the study. Clients either had a low educational level (ie, primary education, prevocational secondary education, years 1-3 of higher secondary education, and vocational secondary education level 1) or intermediate level of education (ie, years 4-6 of higher secondary education and no higher vocational or university education) [31]. Participating members of the social networks were partners (n=1), family members or household members (n=1), and friends (n=2). They also had low or intermediate educational levels and were all current smokers, smoking 5-27.5 cigarettes per day. Nurses were all women and, on average, 53 years of age (SD 10.55) with 9 years (SD 2.61) of experience as a VoorZorg nurse, and 37 out of 97 (38%) of their clients were current smokers (Table 1).

**Table 1:** Sample characteristics of end users.

End users		Age (years), mean (SD)	Stage of change quitting smoking	Number of cigarettes smoked per day, mean (SD)	Planned pregnancy	Age of child (years)	Educational level
**Clients**		24 (4.29)		7.33 (6.29)			
	Client 1		—^a^	—	—	<1	—
	Client 2		Contemplation	20	No	<1	Low
	Client 3		Action	—	Yes	>1	Low
	Client 4		Contemplation	5	No	<1	Intermediate
	Client 5		Precontemplation	—	No	<1	Low
	Client 6		Contemplation	2.5	No	<1	Intermediate
	Client 7		Precontemplation	3.5	Yes	>1	Intermediate
	Client 8		Preparation	6.5	No	<1	Intermediate
	Client 9		Preparation	10	Yes	>1; pregnant	Intermediate
**Social network**		31 (12.42)		19.17 (12.33)			
	Member 1		Contemplation	25	N/A^b^	N/A	Low
	Member 2		—	—	N/A	N/A	—
	Member 3		Precontemplation	27,5	N/A	N/A	Low
	Member 4		Preparation	5	N/A	N/A	Intermediate
Nurses		53 (10.55)	N/A	N/A	N/A	N/A	N/A

^a^Missing data.

^b^N/A: not applicable.

We intended to recruit 8 clients and members of their social networks in design rounds 2, 4, and 5. Approximately half of the recruited participants (n=9) did not show up at these design rounds (phases 2 and 3). On the basis of the standards in the field of usability end user testing [32,33], we intended and recruited 5 end users to evaluate the usability of each interface of Kindle (participation rate 100%; phase 4). Owing to the geographical disparity of end users, some design rounds entailed multiple sessions at different locations. Approximately half of the participants took part in multiple phases, of which 2 nurses participated in two rounds of phase 3 (Table 2).

**Table 2:** Sample participation of end users per design phase and round.

End users			Phase 2				Phase 3						Phase 4
			Round 1		Round 2		Round 3		Round 4		Round 5		Usability
**Clients**													
	Client 1			✓									
	Client 2			✓				✓				✓	
	Client 3			✓				✓				✓	
	Client 4			✓						✓		✓	
	Client 5			✓						✓		✓	
	Client 6							✓					
	Client 7							✓				✓	
	Client 8							✓					
	Client 9									✓			
**Social network**													
	Member 1			✓				✓					
	Member 2			✓									
	Member 3			✓									
	Member 4							✓					
**Nurses**													
	Nurse 1	✓				✓						✓	
	Nurse 2	✓				✓				✓			
	Nurse 3	✓				✓				✓			
	Nurse 4	✓				✓						✓	
	Nurse 5	✓				✓						✓	
	Nurse 6									✓			
	Nurse 7											✓	
	Nurse 8											✓	

#### Procedure Design Sessions

All design sessions of Kindle (ie, phases 2, 3, and 4) with end users took place at easily accessible locations (ie, at Waag, at Youth Health Care Organizations, and at the clients’ home). Each session was prepared and guided by 1 or 2 designers, whereas 1 or 2 researchers observed sessions but did not actively participate in it. After each round, designers, researchers, and the VoorZorg program representative discussed the output of the sessions in a project group. Researchers advised on how to proceed to the next sessions, assimilating the output of the sessions with evidence-based behavior change techniques. The VoorZorg program representative advised on, in conformance with output of the sessions, VoorZorg organizational requirements and opportunities. Thereafter, the designers prepared the next session with end users.

Clients and members of their social environment received a €10 (US $11.8) gift card for their participation in each session; clients received an extra €5 (US $5.9) for every member of their social environment they brought to the session. Organizations were compensated for their nurses’ time participating in the design sessions (€25 [US $29.6] per participating nurse per session). Except for the first design session, this session was part of the nurses’ biennial training.

#### Specification of User Requirements

User requirements were specified in the first two rounds of design inquiry sessions (next to the secondary analysis of our qualitative interviews; phase 1). The first round consisted of nurses (n=51) divided into five user groups. In these sessions, nurse’s perspectives on the requirements of the intervention to be developed were gathered. The session started with brainstorming why their clients quit smoking and relapse. Next, nurses were introduced to game elements and were encouraged to think of game elements that might aid their clients’ smoking cessation or prevent relapse. Finally, nurses were asked to give their perspectives on user requirements other than the inclusion of specific game elements of the intervention to be developed.

The second round was held with clients (n=5) and members of their social networks (n=3). Participants shared their reasons for smoking and alternative activities they could undertake and were encouraged to translate these activities into an app.

### Phase 3: Produce Design Solutions to Meet User Requirements

Preliminary design solutions were created and assessed by end users, starting with an exploration by nurses (n=5; round 3). Next, clients (n=5) and members of their social networks (n=2) assessed the paper mock-up of Kindle (round 4). Finally, an improved paper mock-up was assessed by both nurses (n=3) and clients (n=3; round 5).

### Phase 4: Evaluate Against Requirements

#### Heuristic Evaluation

A total of 3 human factors engineering experts received a guideline containing background information about VoorZorg, why Kindle was developed, its end users, and aims. Moreover, the guideline explained how to install and open the app on smartphones and entailed instructions on performing the usability inspection. No training was given to the experts on how to use the Kindle before the heuristic evaluation. We instructed the experts to systematically evaluate both the nurse and client interfaces of Kindle by freely exploring the functionalities. Experts were asked to describe usability flaws in detail and classify them according to the HIMSS principles [28]. Experts were instructed to rate the severity of the problems they encountered according to the Nielsen five-point Likert severity rating (0 indicating no usability problem to 4 indicating a usability catastrophe) [34]. Experts were encouraged to write comments to further explain the rationale for their ratings.

The results of the usability inspections by all 3 experts were merged according to functionality and HIMSS principles [28]. The average severity score was calculated when multiple experts identified the same usability problem.

#### Think Aloud Method

All end users (ie, nurses and clients) had prior experience with smartphone apps and were informants during the design phase of Kindle. However, none of the participants had prior experience with the prototype. Data collection lasted 20-50 minutes and took place at clients’ homes and at the nurses’ workplace at Youth Health Care Organizations. Participants used the smartphones of the evaluators to perform the usability test of Kindle, which was video recorded via a third-party smartphone app (ie, AZ Screen Recorder [by Hecorat Global Technology], downloaded from the Google Play Store that recorded the smartphone screen, audio, and user inputs). We explained to participants that they would use the app by performing specific tasks provided by an evaluator (MD or SS). Participants were instructed to verbalize their thoughts while performing the tasks. Before the actual usability test, participants were given a warm-up task to practice thinking aloud. The warm-up task was to add a specific contact and contact details to the contacts list while thinking aloud. None of the participants had difficulty verbalizing their thoughts during the warm-up task.

The tasks of the usability test were based on real-life scenarios in which testing of all the main functions of the app was covered. The series of tasks were always conducted in a fixed order across participants (Textbox 1). The evaluator reminded participants to continue thinking aloud when they stopped doing so. If a participant was not able to complete a task after three attempts, the evaluator provided a clue.

**Think aloud usability tasks.**

**Nurse Interface**

- Create profile
- Create group
- Manage group
- Manage personal goals of clients
- Use chat functionalities
- Read and add tips (ie, advice)

**Client Interface**

- Create profile
- Create personal goals
- Use chat functionalities
- Read and add tips (ie, advice)
- Use personal diary

To reveal and describe usability issues in detail, one of the think aloud evaluators (SS) analyzed the videos by coding participants’ utterances and user input per task (ie, functionality). A usability issue was reported when a participant was not able to complete the instructed task in her first attempt. The reported usability problems were then categorized according to the HIMSS usability principles by a think aloud evaluator (SS) [28]. Next, the think aloud protocols were merged by the end user group to provide an overview of usability problems per functionality. These were then merged with the findings from the heuristic evaluation, after which all usability problems from the heuristic evaluation and think aloud method were discussed, and recommendations were made to resolve each usability problem (SS, MD, and MJ).

## Results

### Outcomes Phase 1: Understanding and Specification of the User Context

A secondary analysis of qualitative interview data revealed that clients generally indicated good relationships with their VoorZorg nurse. During home visits, clients and nurses kept in touch via WhatsApp, with varied intensity (eg, some clients and nurses only communicated concerning appointments and others would regularly ask personal and medical questions via WhatsApp). Most clients interviewed did not have a job nor were they currently enrolled in education. A number of clients did not live on their own but, for example, lived with their parents or in assisted living facilities. Clients had limited social networks and were normally not in contact with other VoorZorg clients. All interviewed clients had a smartphone and access to the internet. Only a few clients had a tablet, laptop, computer, or game console.

### Outcomes Phase 2: Specification of User and Organizational Requirements

The user and organizational requirements that we identified during the first two rounds of Kindle’s design sessions with nurses, clients, and clients’ social networks were divided into design requirements and functionality requirements (Textbox 2).

**User and organizational requirements.**

**Design**

- Mobile health app
- Easy to use, simple use of language, little use of texts, and visualizing content due to lower health literacy of clients
- Social media–like design
- Not necessarily be presented as smoking cessation intervention
- Harmonizing VoorZorg values (ie, no advising, judging, patronizing, pedantic tone, and yet following and endorsing clients)
- App with multiple functionalities to digitalize aspects of the VoorZorg program
- Usable for both nurses and clients
- Nonaddictive or time consuming
- No costs for clients
- Not childish
- Positive focus

**Functionalities**

- Enabling secured communication between nurses and clients (ie, social support)
- Enabling anonymous communication between clients (ie, peer contact)
- Tailored at early stages of change in smoking cessation (ie, precontemplation, contemplation, and preparation [23])
- Focus on gaining control over life
- Providing a way of dealing with stressors and boredom
- Arousing intrinsic motivation for smoking cessation
- Providing clients with self-understanding and building self-efficacy in clients (ie, social support)
- Rewarding and acknowledging clients’ efforts (ie, social support and game element)
- Challenging (game element)
- Earning points or compliments efforts (ie, social support and game element)
- Providing information

### Outcomes Phase 3: Production of Design Solutions to Meet User and Organizational Requirements

**Figure 1:**
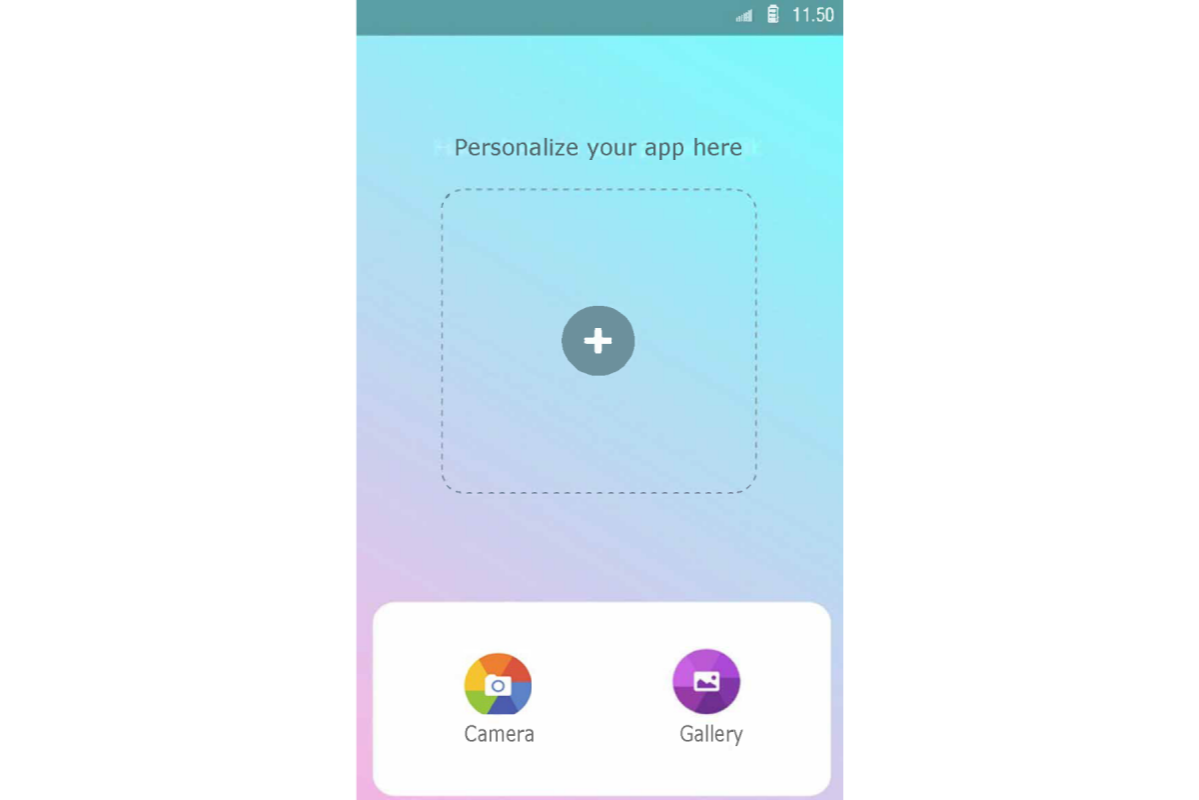
Example screenshot of the profile creation section where the user can choose an avatar (identical in both interfaces).

**Figure 2:**
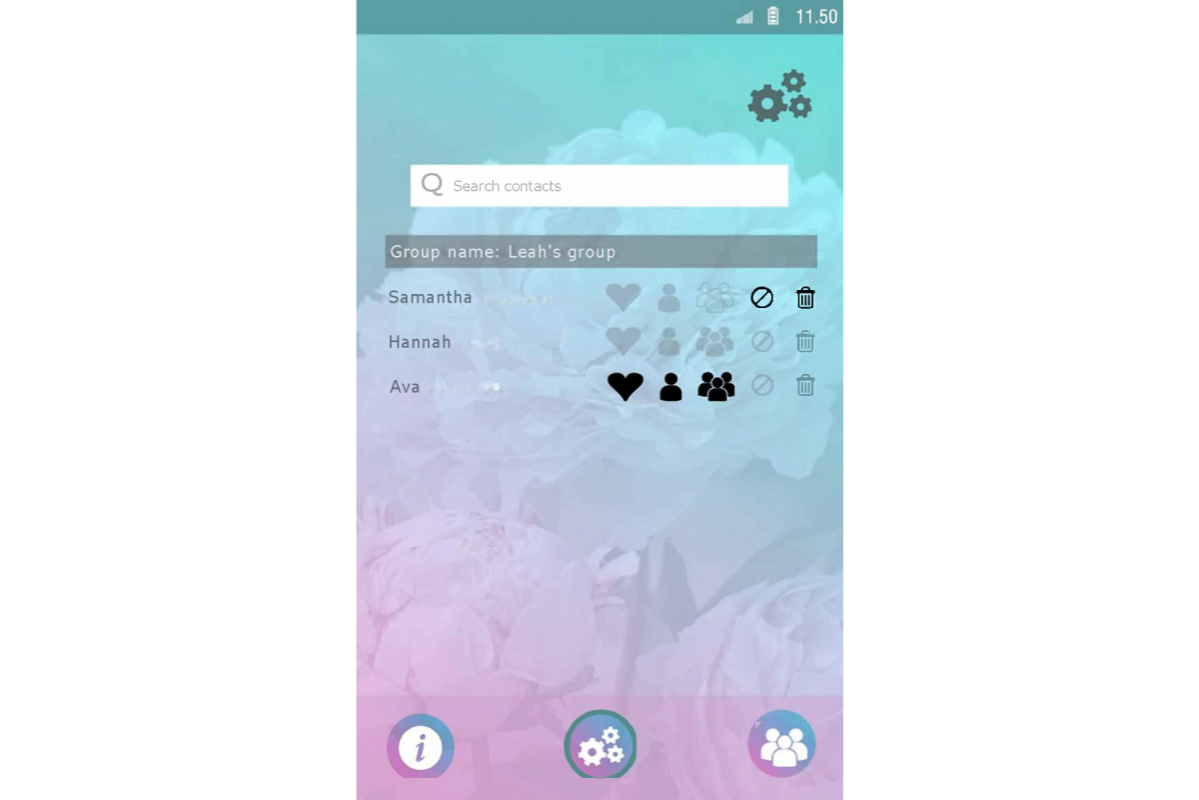
Example screenshot of the admin panel in the nurse interface of Kindle.

**Figure 3:**
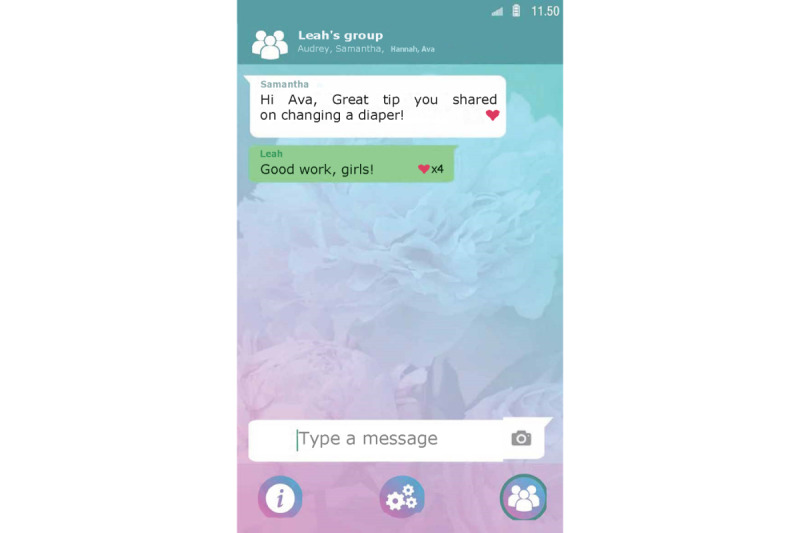
Example screenshot of the group chat functionality in the nurse interface of Kindle.

**Figure 4:**
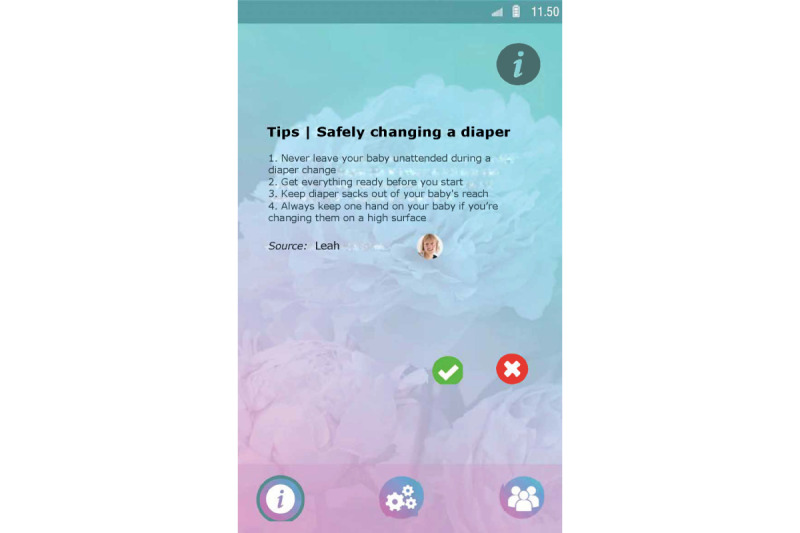
Example screenshot of the tip functionality of the nurse interface of Kindle.

**Figure 5:**
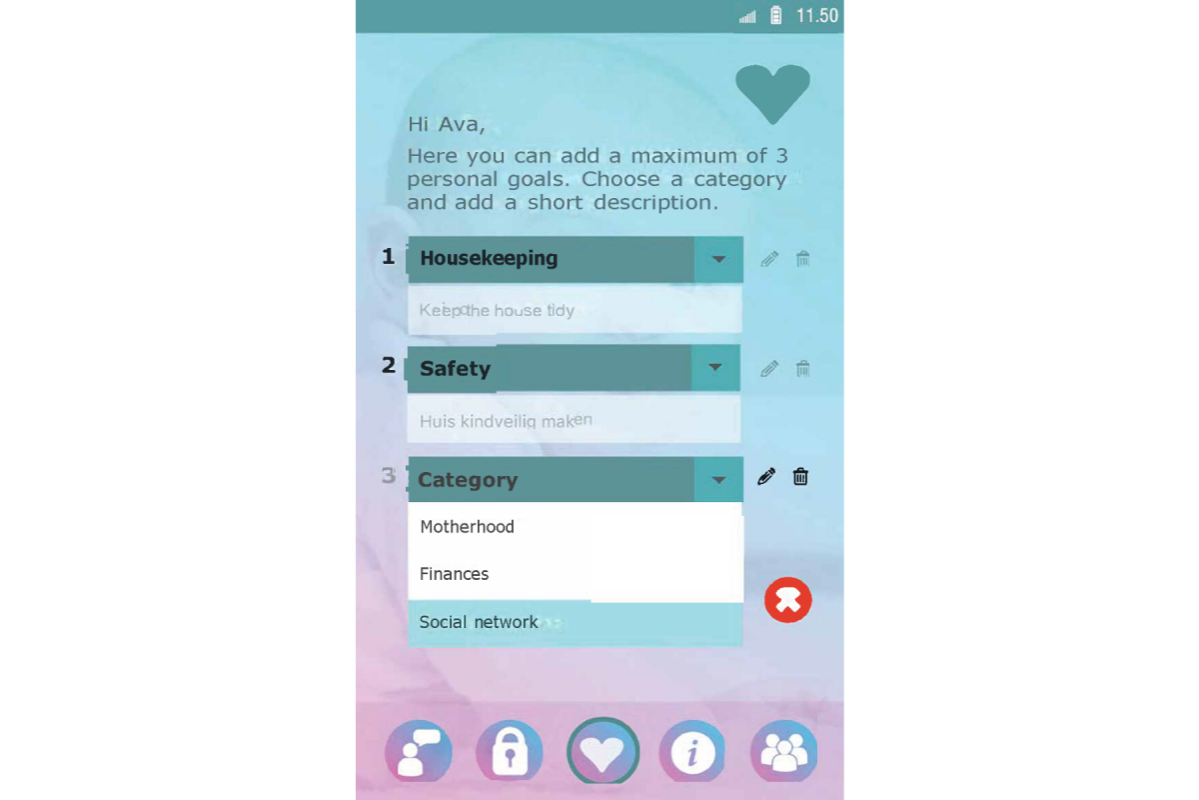
Example screenshot of the goal setting functionality in the client interface of Kindle.

**Figure 6:**
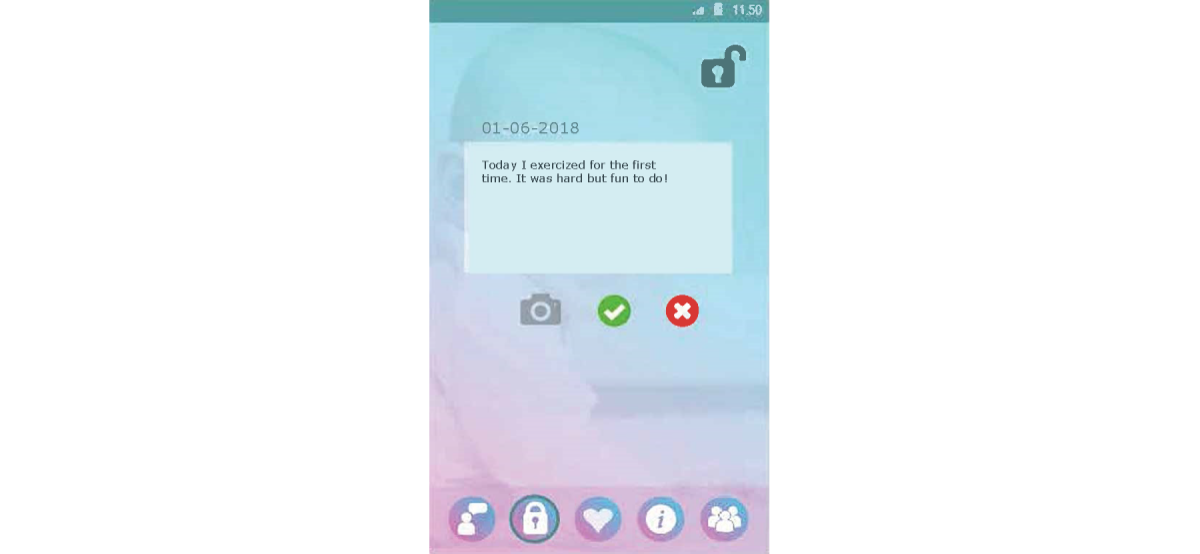
Example screenshot of the diary functionality of the client interface of Kindle.

The design sessions resulted in a preliminary prototype, named *Kindle* (Textbox 3; Figures 1-6), meeting all user and organizational requirements.

**Intervention characteristics of Kindle.**

**Name**

- Kindle

**Type of Intervention**

- Mobile health app

**Aim**

- To support women through the first stages of smoking cessation

**Targeted Determinants**

- Increasing clients’ readiness for smoking cessation
- Creating a supportive social network for clients
- Increasing clients’ self-efficacy in obtaining personal goals
- Increasing clients’ knowledge and self-efficacy (eg, tips)
- Improving communication with nurse (eg, secured chatting)
- Processing difficulties in life (eg, diary)

**Setting**

- Developed for use in a care setting and at clients’ home

**Nurse Interface Functionalities**

- Set up a profile by entering their name and choosing an avatar representing themselves and choosing from taking a picture with their smartphone camera or an image from their smartphone gallery (Figure 1).
- Manage clients from the admin panel. Nurses can add and delete clients to and from Kindle. Moreover, nurses can block clients from participating in the group chat (Figure 2).
- Endorse and reward clients for their progress in obtaining their goals by assigning hearts (ie, heart-shaped points).
- Communicate with clients via secured private chat and group chat (Figure 3; ie, secured server). All messages in the chat functionality could be loved by tapping a heart-shaped button (ie, similar to the “like” functionality on social media), by which clients were empowered in their contributions to the chat.
- Create tips or moderate tips shared by clients (Figure 4).

**Client Interface Functionalities**

- Set up a profile by entering their name and choosing an avatar representing themselves and choosing from taking a picture with their smartphone camera or an image from their smartphone gallery (Figure 1).
- Formulate personal goals (ie, “heart desires”), by which they could work on resolving barriers for smoking cessation and build self-efficacy in obtaining personal goals. Women can select a category (ie, being a mother, healthy lifestyle, my child, work and leisure, safety, finances, talking and listening, family and friends, and help) and then enter their personal goal (Figure 5). Clients could enter three active personal goals to work on at the same time.
- View their personal goal attainment progress (ie, 50 hearts represent an obtained goal).
- Communicate with nurse via secured private chat and group chat. All messages in the chat functionality could be “loved.”
- Read and create tips by and for other clients.
- Write private posts in their digital diary; clients could also add images to their posts (Figure 6).

**Game Elements [35]**

- Avatar creation (ie, setting up profile)
- Player management features (ie, personal goals and progress)
- Intermittent rewards (ie, earning hearts with progress in personal goals)
- Social utility (ie, tips)
- Support network (ie, chat)
- User input (ie, tips and diary)

**Development Stage**

- Early—the prototype had limited functionality, being a series of screenshots that were linked together via clickable buttons

### Outcomes Phase 4: Evaluation Against Requirements

#### Usability Problems Within the Nurse Interface of Kindle

**Figure 7:**
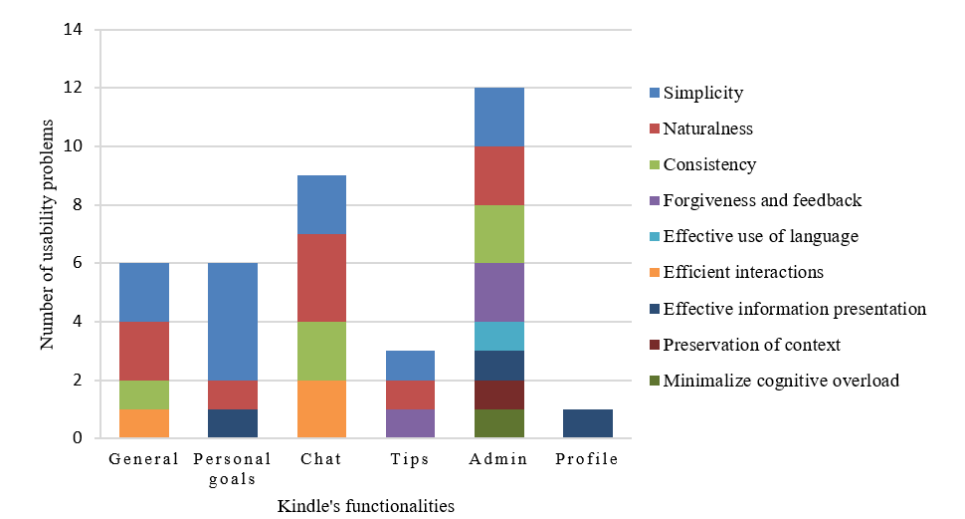
The number of usability problems within the nurse interface per functionality and principle.

We found 37 usability problems within the nurse interface of Kindle (Figure 7). We identified the general problems and problems related to functionalities. Most usability problems (n=12) were found in the admin functionality (eg, issues of consistency—using the same icon or button with different meanings), followed by the chat function (n=9; eg, issues for efficient interactions—it is unclear when messages are sent). Most usability issues revealed by both evaluation methods concerned violation of the simplicity of the HIMSS principles (eg, the private chat function is hidden in the admin menu) and naturalness (eg, unclear icons). In total, 24 of 37 (65%) potential usability problems were detected in the heuristic evaluation, 7 of 37 (19%) usability problems were detected in the think aloud method, and 6 of 37 (16%) usability problems were detected by both heuristic evaluation and think aloud. The mean severity of usability problems detected through heuristic evaluation was rated rated as 1.8 (SD 1.00), reflecting that the usability problems found by experts were, on average, minor. We provide a complete overview of the usability problems of the nurse interface of Kindle, per the HIMSS principle found through heuristic evaluation and think aloud and provide a recommendation to solve the issue (Multimedia Appendix 1, Table S1).

#### Usability Problems Within the Client Interface of Kindle

**Figure 8:**
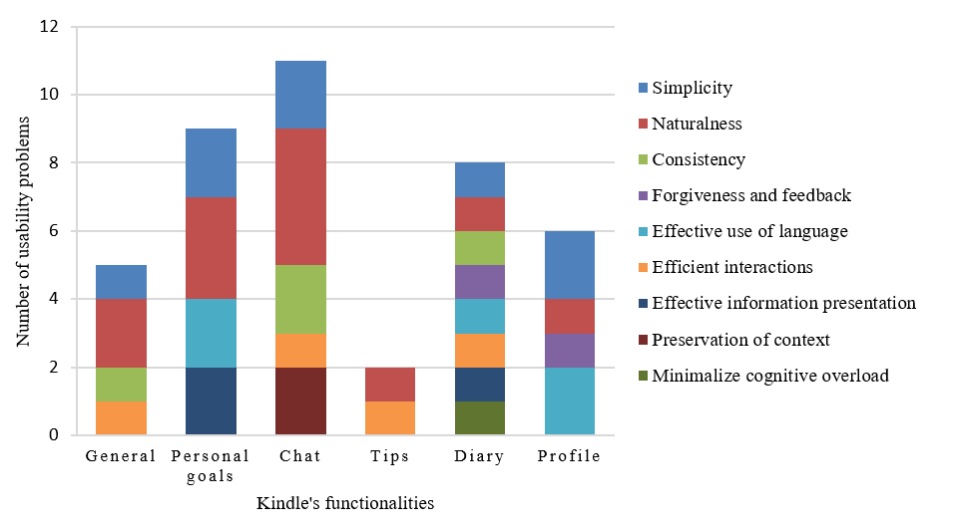
The number of usability problems within the client interface per functionality and principle.

In total, 41 usability problems within the client interface of Kindle were discovered (Figure 8). We identified general problems and problems based on their functionality. Most usability problems (n=11) were found in the chat functionality (eg, issue of consistency—the group chat does not have the heart icon next to the input field, unlike the private chat), followed by the personal goals function (n=9; eg, issue of simplicity—the numbers above the golden heart icons are unclear). Most usability issues by both evaluation methods concerned violation of the HIMSS principles simplicity (eg, it is not clear that the heart is a clickable button to give a *like*) and naturalness (eg, it is not clear that the lock icon in the navigation bar represents a diary). In total, 31 of 41 (76%) potential usability problems were detected in the heuristic evaluation, 4 of 41 (10%) usability problems were detected in the think aloud method, and 6 of 41 (15%) usability problems were detected by both heuristic evaluation and think aloud. The mean severity of usability problems detected through heuristic evaluation was rated as 1.8 (SD 0.81), reflecting that the usability problems found by experts were minor. A complete overview of the usability problems of the client interface of Kindle, per the HIMSS principle found through heuristic evaluation and thinking aloud, and a recommendation to solve the issue can be found in Multimedia Appendix 1, Table S2.

#### Recommendations to Improve the Usability of Kindle

**Figure 9:**
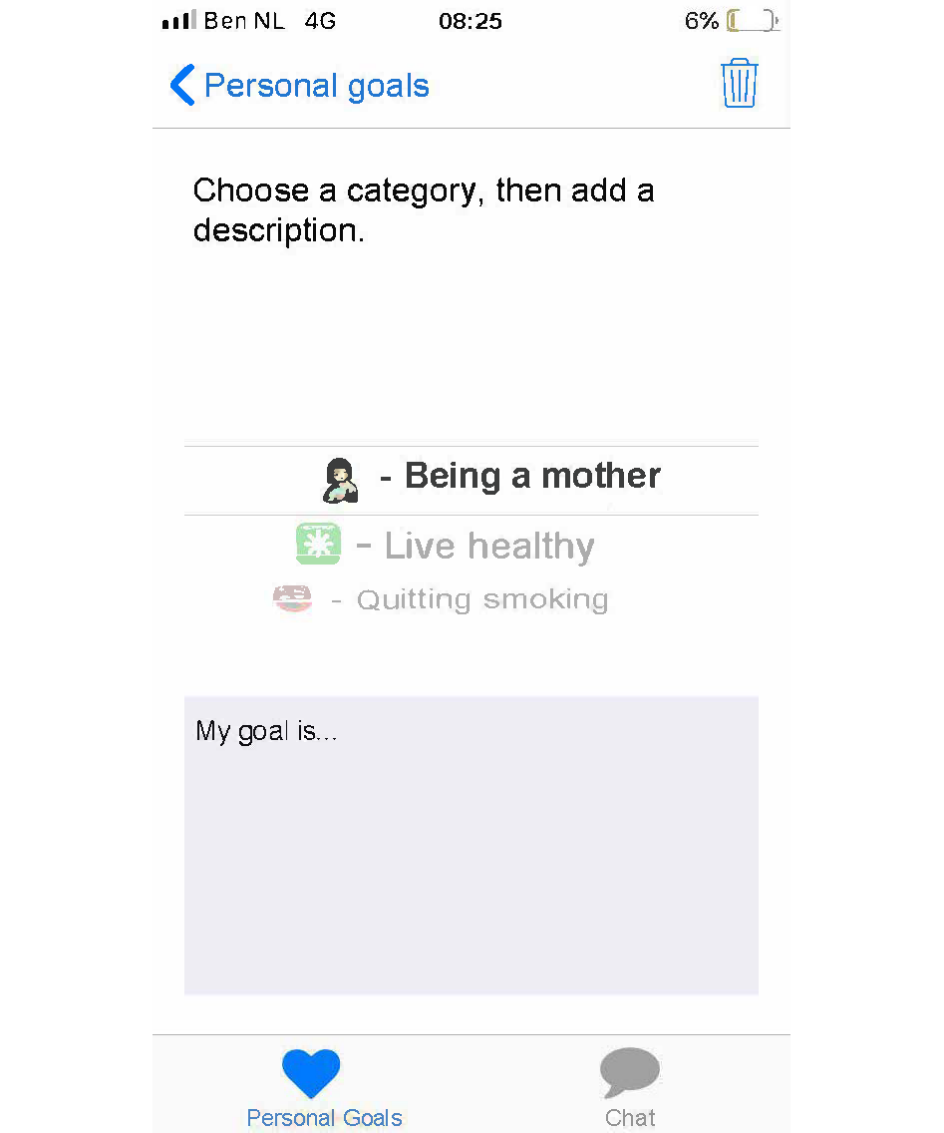
Example screenshot of the goal setting functionality in the client interface of Kindle.

**Figure 10:**
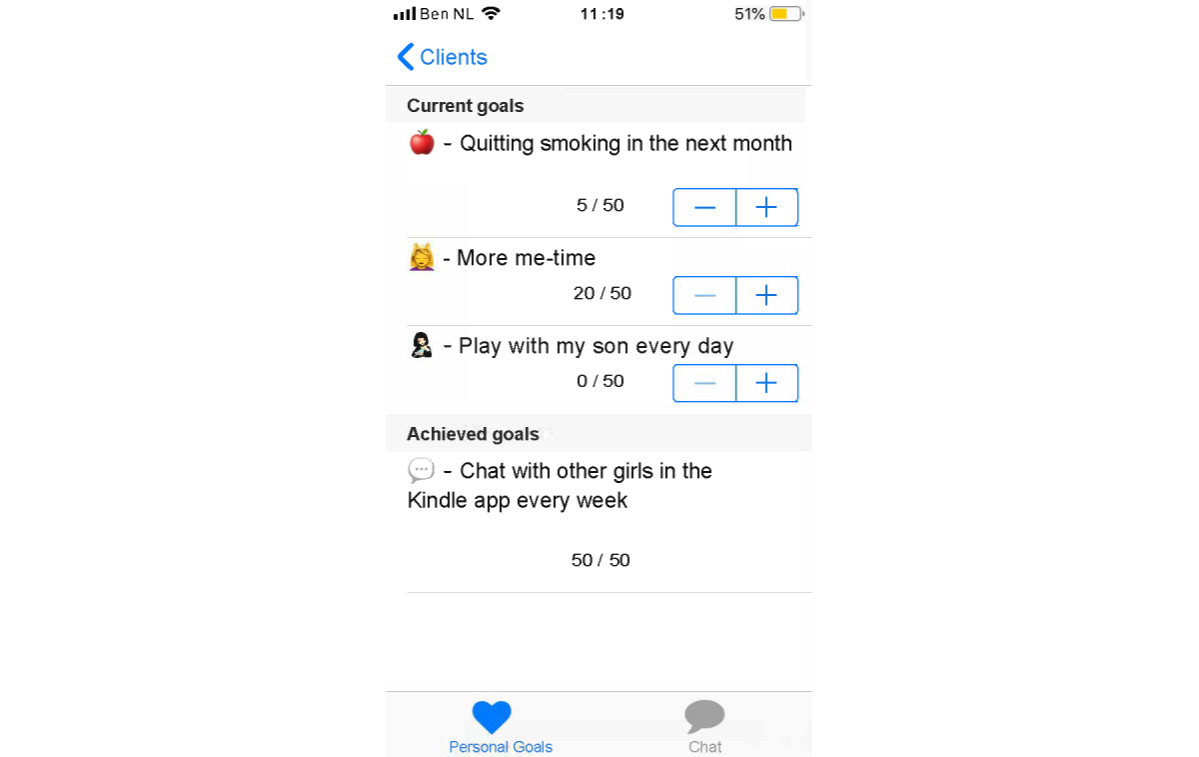
Example screenshot of the goal setting functionality in the nurse interface of Kindle.

**Figure 11:**
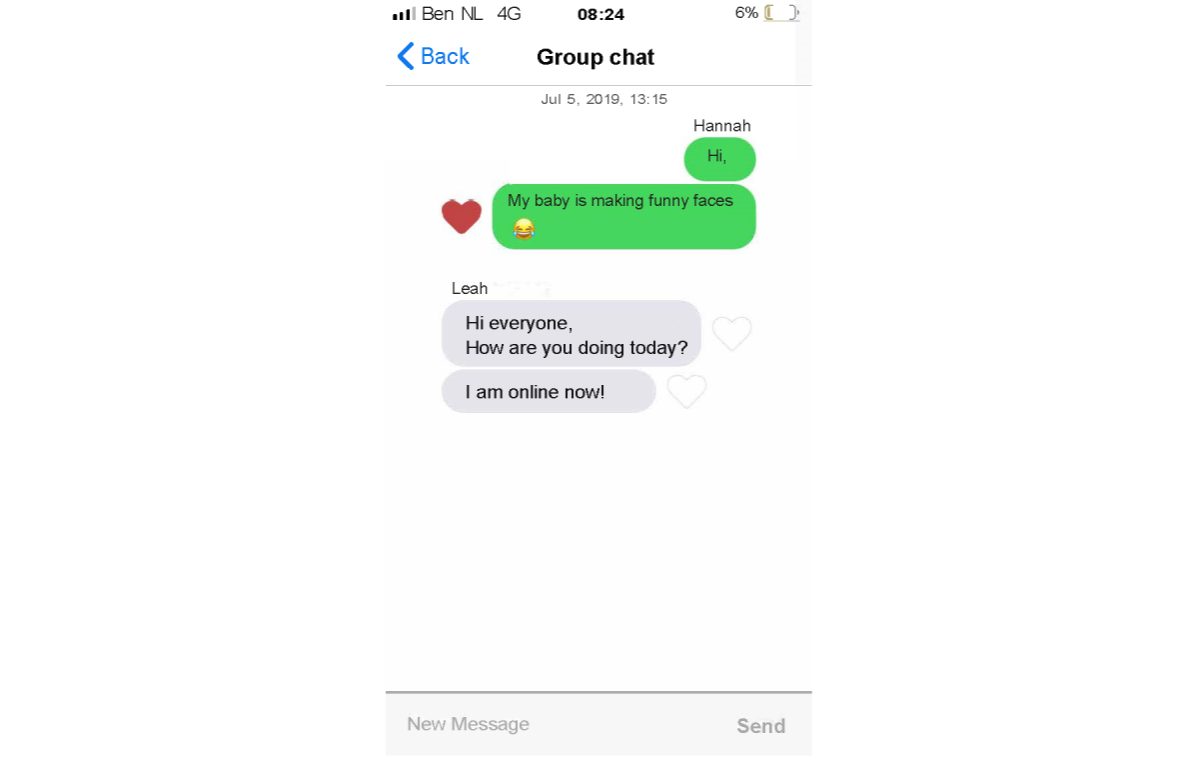
Example screenshot of the group chat functionality in the client interface of Kindle.

For each usability problem, researchers SS and MD and human factors engineering expert MJ made recommendations to improve the usability of the nurses and client interface of the Kindle prototype (Multimedia Appendix 1, Tables S1 and S2). A final iteration round following the recommendations resulted in a final version of Kindle (Textbox 4; Figures 9-11). This final version was evaluated in a pilot study.

**Final version of intervention characteristics.**

**Name**

- Kindle

**Intervention Type**

- Mobile health app

**Aim**

- To support women through the first stages of smoking cessation

**Targeted Determinants**

- Increasing clients’ readiness for smoking cessation
- Creating a supportive social network for clients
- Increasing clients’ self-efficacy in obtaining personal goals
- Improving communication with nurse (eg, secured chatting)

**Setting**

Developed for use in a care setting and at clients’ home

**Nurse Interface Functionalities**

- Manage clients from the admin panel. Nurses can add and delete clients to and from Kindle. Moreover, nurses can block clients from participating in the group.
- Endorse and reward clients for their progress in obtaining their goals by assigning hearts (ie, heart-shaped points).
- Communicate with their clients via a secured private chat and group chat (ie, secured server). All messages in the chat functionality could be “loved” by tapping a heart-shaped button (ie, similar to the “like” functionality on social media), by which clients were empowered in their contributions to the chat.

**Client Interface Functionalities**

- Formulate personal goals (ie, “heart desires”), by which they could work on resolving barriers for smoking cessation and build self-efficacy in obtaining personal goals. Women can select a category (ie, being a mother, healthy lifestyle, my child, work and leisure, safety, finances, talking and listening, family and friends, and help) and then enter their personal goal.
- View their goal attainment progress (ie, 50 hearts represent an obtained goal).
- Communicate with their nurses via a secured private chat and in a group chat with peers (ie, other clients). All messages in the chat functionality could be “loved.”

**Game Elements [35]**

- Player management features (ie, personal goals)
- Intermittent rewards (ie, earning hearts with progress in personal goals)
- Support network (ie, chat)

**Development Stage**

- Advanced: fully functional for pilot implementation and evaluation.

## Discussion

### Principal Findings

In this paper, we describe the user-centered design and usability evaluation of an mHealth app (*Kindle*) that supports disadvantaged young women during and after pregnancy by moving through the first stages of smoking cessation. Disadvantaged women, members of their social networks, and nurses were informants throughout the phases of the iterative prototype design. In the first phase of the intervention design, secondary analysis of qualitative interview data revealed that nurses and clients keep in touch through WhatsApp during home visits and that all interviewed clients had smartphones and internet access but usually possessed no other devices. The clients were not in contact with other clients. In phase 2, we established user and organizational requirements from the secondary interview data and design sessions with end users. The main requirement was that the intervention should be an mHealth app, offering secure communication between nurses and clients. Moreover, the intervention should be tailored to the early stages of change in smoking cessation, include game and social support elements, and have easy-to-use interfaces. In phase 3, the Kindle prototype with game elements was developed technically. Kindle combines a nurse and client interface and includes the following functionalities: personal goal setting with earning points, chat function with a nurse and other clients, tips, and admin function or diary and profile creation. Prototype usability (phase 4) was evaluated by a combination of heuristic evaluation among experts and think aloud sessions among end users (ie, nurses and disadvantaged women). We found 78 usability problems for both interfaces. Most usability problems concerned violation of the principles of *simplicity* and *naturalness* and were found in the chat (both interfaces), admin (nurse interface), and goal setting (client interface) functionalities. Following the recommendations from the usability evaluation, a final iteration round resulted in a final version of Kindle.

### Comparison With Prior Work

The first phase of our user-centered design was devoted to understanding and specifying the user context, resulting in a specific focus on the early stages of smoking behavior change [23]. This is in contrast to action stage–oriented smoking cessation apps, which are widely available or being developed [7,36,37]. The use of the transtheoretical model in interventions is associated with positive effects on health behavior [38]. According to an inventory by Paige et al [37], processes of change that aid people in moving through stages of behavioral change are widely applied in mHealth apps for smoking cessation, including Kindle. Moreover, in accordance with other research [8,39-42], the first phase highlighted the importance of supportive social networks for smoking cessation.

By involving both end user groups (clients and nurses), we were able to identify key user and organizational requirements (ie, phase 2) and to incorporate them into Kindle’s design solution (ie, phase 3). This coherence of the mHealth app with user objectives or requirements has been identified as one of the critical factors for smoking cessation mHealth apps [43]. Moreover, we involved end users as informants in our design process, which has been found to be more effective in changing behavioral determinants [14]. The involvement of socioeconomically disadvantaged populations thus appears to be a feasible and effective strategy in mHealth design.

One of the user requirements of Kindle that we identified was the use of game elements and social interactions. Previous research has also suggested the potential of social features in serious games for smoking cessation [8]. Moreover, pregnant women in other studies also highlighted the usefulness and playfulness of social interaction functionalities within mHealth [10]. Yet, among smoking cessation mHealth apps, few have a game or social nature similar to that of Kindle [37,44,45]. Kindle further comprises a unique, secured chat functionality, whereas only 16% of mHealth smoking cessation apps integrated web-based communication exclusive to the app [37]. This is striking because peer-to-peer communication and communication with an advisor (eg, health care professional) is associated with more effective eHealth interventions [38,46] and is generally highly preferred among pregnant women in web-based apps [10].

Goal setting and rewards were included as game elements in Kindle. These functionalities are also regularly found in smoking cessation mHealth apps [44,47] and appear to have a significant positive impact on health behavior [38]. However, only 15% of mHealth apps for smoking cessation have a progress tracking feature similar to that of Kindle [44]. As we did not find evidence on the effectiveness of functionalities or game elements concerning sharing or creating tips and keeping a digital diary, these elements were not incorporated in the final version of Kindle.

Aligning interventions to the low health literacy levels of clients was another user and organization requirement. Similar to Kindle, most mHealth apps for smoking cessation incorporated plain usage of language as part of health literacy considerations [37].

Finally, it was also a user and organizational requirement that the app be free of charge for end users. Kindle will be freely available, similar to many other smoking cessation mHealth apps [37,44,45,47].

In our usability evaluation, human factors engineering experts inspected and end users tested for each interface (ie, clients and nurses) of Kindle through heuristic evaluation and think aloud, respectively, which has now become a general practice in usability evaluations [48]. The types of problems detected in our study differed according to each evaluation method. The think aloud method with end users disclosed more critical usability problems, concerning being able to actually use the app as intended, whereas the heuristic evaluation among experts mainly resulted in the disclosure of less severe, noncritical problems, concerning the ease of use of the app or a less optimal user experience. These findings are in accordance with earlier research [49,50] and demonstrate that the combination of expert and user usability methods was truly complementary, and result in surplus value in the design of a usable app [26,49].

Most usability problems were found in the chat functionality of both interfaces, the admin function in the nurse interface, and goal setting in the client interface and concerned *simplicity* and *naturalness* issues. We formulated recommendations to resolve these issues, so that Kindle, similar to other smoking cessation mHealth apps, obtains good scores on functionality and esthetics [45]. According to participants in co-designing a self-management mHealth intervention, an app’s usability and intuitiveness might be negatively affected by the inclusion of numerous functionalities [51]. Consequently, in the final iteration round, Kindle is expected to benefit from fewer functionalities, whereas the remaining ones (ie, goal setting and chat) should follow usability standards.

Usability evaluations among disadvantaged populations are scarce [12]. However, disadvantaged populations may reveal unique usability problems in terms of the content and functionalities of interventions [52]. This was also reflected in our study, where disadvantaged women revealed approximately 10% (4/41) of the usability problems with the client interface. Nurses detected more usability problems (approximately 7/37, 19%), yet these were mainly found in the admin function, which was not a functionality of the client interface. This relatively low number of problems might be a positive side effect of our user-centered approach to the design of Kindle, which is expected to resolve potential usability issues in early stage versions of the intervention.

### Strengths and Limitations

A strength of our study was the involvement of end users throughout all phases of the user-centered design and usability evaluation of Kindle. The involvement and input of disadvantaged women in the design sessions were highly valuable. In this way, we were able to meet their (and organizational) requirements. This will likely result in higher acceptance of the implementation of Kindle as an intervention and, consequently, is expected to support its effectiveness. Another strength of our study was the triangulation of methods in the fourth phase of Kindle’s design. We used two types of usability evaluation methods to detect usability problems in our prototype, which provided us with a more complete overview of usability problems, as only approximately 15% (6/37 and 6/41) of the usability problems found by think aloud and heuristic evaluation overlapped.

Our study also had limitations. First, the design sessions were attended by fewer clients than intended and recruited. The clients often did not show up to a session they had confirmed to attend. Involving disadvantaged populations in research is challenging [5,53]. Moreover, with less than 1% of the births per Dutch municipality qualifying to enroll in VoorZorg, our target population is very small. Nonetheless, we succeeded in fulfilling multiple rounds of design with mixed compositions of participants. In qualitative health research and usability end user tests, smaller sample sizes are acceptable, as they provide higher information power [32,33,54].

Another limitation was the involvement of a limited number of members of women’s social networks in most rounds of intervention design. Identical to clients’ no shows, we were not able to recruit as many members of social networks as intended. However, during Kindle’s design process, we found that existing social networks mainly had a negative role in clients’ smoking cessation efforts [22], and clients wanted support from peers.

Moreover, we evaluated the usability of a prototype with limited functionality. The think aloud method was based on certain real-life tasks that covered all the functionalities. This may have highlighted other usability problems that would have occurred during free use of the app. The limited functionality of the prototype may also have resulted in an incomplete insight into usability problems. However, conducting a usability evaluation using heuristic evaluation and think aloud is common in early system design phases, as insights can be used to redesign the system [26].

### Practical and Research Implications

Our study adds to the limited existing research following and reporting on all phases of user-centered design of mHealth interventions aimed at disadvantaged populations and a small fraction of studies that report the results of their usability evaluation [48]. Our study indicates that disadvantaged women are capable of participating in all phases of the intervention design. Their input has been valuable in detecting their needs and important usability problems while performing tasks to evaluate Kindle’s usability. However, the attendance of disadvantaged women in the design sessions was less than intended and recruited. This implies that more research is needed to gain insight into how disadvantaged populations can be involved in all user-centered design processes and usability evaluations of mHealth interventions aimed at these populations. This may help achieve improved intervention reach, adoption, and implementation among disadvantaged populations.

Another research implication stems from the attendance of multiple clients in the design sessions. This showed the added value of connecting clients with other clients (ie, peers), rather than involving existing social networks of women in the intervention design. The social interactions were positive, supported clients, and inspired both end users and designers to incorporate aspects of these interactions in the design solutions. More research is needed on the effectiveness of such social components of digital interventions on health behavior change.

Practically, we will use the results of this study to pilot test Kindle in the VoorZorg context. Finally, we aim to implement Kindle in the nationwide VoorZorg program and other Dutch care settings that encompass intensive support from health care professionals with disadvantaged clients.

### Conclusions

The user-centered design and usability evaluation of Kindle provided valuable insights for improving its first design. By involving health care professionals and socioeconomically disadvantaged, young women during and after their pregnancy (ie, end users), we were able to gain insight into their context, needs, and requirements. Consequently, together with the end users, we were able to meet their requirements to achieve readiness for smoking cessation in our first design solution. We evaluated the usability of the prototype through experts and end users, which revealed unique usability problems for this population. These insights allow for further optimization of Kindle, and we encourage future studies to engage disadvantaged populations in mHealth intervention design and usability testing.

## Appendix

Multimedia Appendix 1 Overview of usability problems per user interface.

## Abbreviations

HIMSS: Healthcare Information and Management Systems Society
mHealth: mobile health
