# Supplementary material for: Mobile Health for Smoking Cessation Among Disadvantaged Young Women During and After Pregnancy: User-Centered Design and Usability Study
Source: JMIR Form Res. 2021 Aug 4;5(8):e24112. doi: 10.2196/24112 (PMC8374659; doi:10.2196/24112)
Supplement: Multimedia Appendix 1 [file formative_v5i8e24112_app1.docx]

## Multimedia Appendix I

Table S1. Nurse interface usability evaluation results

| Functionality | Heuristic principle | Usability problem | Detection | Severity^a^ | Recommendation |
| --- | --- | --- | --- | --- | --- |
|  |  |  |  |  |  |
| General |  |  |  |  |  |
|  | Simplicity |  |  |  |  |
|  |  | Experts found it unclear if you can have multiple groups as a nurse. | Heuristic evaluation: 1/3 experts  Think aloud: 0/5 end users | 3 | Inform the end user how many client-groups can be created. |
|  |  | It is confusing to use multiple icons in order to show in which menu the user finds itself.  Heuristic Evaluation expert(s): *“There is a highlighted icon in the navigation bar of the corresponding page, and this same icon (which is non-clickable, but looks clickable) is also put in the right upper corner of the screen.”* | Heuristic evaluation: 2/3 experts  Think aloud: 0/5 end users | 1.5 | Only use the highlighted icon in the navigation bar, and (if necessary) use a title bar with the name of the menu on top. |
|  | Naturalness |  |  |  |  |
|  |  | User prompts are ambiguous and are not explicit enough.  When deleting a client from the group, the end user had problems finding the correct button to “accept” the removal, and pressed the “cancel” button instead. | Heuristic evaluation: 2/3 experts  Think aloud: 1/5 end users | 3.5 | Replace all the green-check-icons and red-cross-icons for “Proceed”, “Go back”, “Save”, “Delete” buttons etcetera to make it more explicit to the user what the button actually does in this context. |
|  |  | The icons are not intuitive and cause confusion. | Heuristic evaluation: 2/3 experts  Think aloud: 0/5 end users | 3.5 | Add text to the icons to improve understandability, and check material.io for guidelines on how to create better understandable icons. |
|  | Consistency |  |  |  |  |
|  |  | The placement of icons, which show the current page, differs per page. The Tips and admin icon are placed in the upper right corner, while the group-chat icon is placed in the upper-left corner. | Heuristic evaluation: 3/3 experts  Think aloud: 0/5 end users | 1 | Pick a spot on screen for the icons, and stay consistent. |
|  | Efficient interactions |  |  |  |  |
|  |  | Dismissing the keyboard is only possible by clicking “return”. Clicking on the screen does not work, although this works in all other apps. | Heuristic evaluation: 0/3 experts  Think aloud: 2/5 end users | N/A | Let the user dismiss the keyboard by pressing on a spot on the screen, which is not the keyboard. |
| Personal goals |  |  |  |  |  |
|  | Simplicity |  |  |  |  |
|  |  | The “personal goals” function was not intuitive to access by the end users.  Expert(s): “*A basic functionality like personal goals should not be put away in the admin menu.*” | Heuristic evaluation: 1/3 experts  Think aloud: 2/5 end users | 4 | Make personal goals accessible through a dashboard page. |
|  |  | It is not clear that the user can give hearts to their clients’ “personal goals” by clicking on the golden heart. | Heuristic evaluation: 2/3 experts  Think aloud: 2/5 end users | 3 | Emphasize that a heart can be given by using text, or a “plus” sign. |
|  |  | It is not clear that “completed personal goals” list the personal goals that are already completed. | Heuristic evaluation: 0/3 experts  Think aloud: 2/5 end users | N/A | It could be that the word “completed” is hard to understand for end users. If that is the case, a synonym like “achieved” could be used.  Another cause for the problem could be that, the way that the “current personal goals” is presented, is too similar of that of “completed personal goals”, and therefore end users do not notice the difference between the pages. If this is the case, a different layout could be used to emphasize the difference between the pages. |
|  |  | In the “current personal goals” section it is not clear that the goal amount of hearts is 50. | Heuristic evaluation: 0/3 experts  Think aloud: 1/5 end users | N/A | A possible solution is adding a denominator of “50” to the amount of hearts for each personal goal. |
|  | Naturalness |  |  |  |  |
|  |  | The golden-heart-rating-system is unclear.  Expert(s): “*Who assigns the ratings? The nurse or the client? Couldn’t figure it out after seeing both the nurse and client app.”* | Heuristic evaluation: 2/3 experts  Think aloud: 0/5 end users | 3 | A short description would help. E.g. “Here you can score Eva based on the following criteria.”. |
|  | Effective information presentation |  |  |  |  |
|  |  | The participant did not notice the heart-counter increased when tapping on the “give-heart” button. Therefore the participant thought the button did not work | Heuristic evaluation: 0/3 experts  Think aloud: 1/5 end users | N/A | A small animation emphasizing that the heart-counter went up could be implemented. |
| Chat (general) |  |  |  |  |  |
|  | Consistency |  |  |  |  |
|  |  | Both user interfaces are inconsistent in how to open the chat functionality.  The nurse opens a private chat by clicking on the “pawn” icon from the admin section, while the client opens a private chat from the “pawn” icon in the navigation bar below. | Heuristic evaluation: 2/3 experts  Think aloud: 0/5 end users | 3 | Although an end user is either a client or a nurse, and will therefore not use both interfaces, it could be advantageous to have a similar navigation structure across both interfaces. |
|  | Efficient interactions |  |  |  |  |
|  |  | There is no search functionality in chats.  Heuristic Evaluation expert(s):*”Users should be able to search for keywords in the chat for efficient information retrieval”.* | Heuristic evaluation: 2/3 experts  Think aloud: 0/5 end users | 1 | Include a search functionality in the chat. |
|  |  | It is unclear when messages were sent. | Heuristic evaluation: 2/3 experts  Think aloud: 0/5 end users | 1.5 | Include a “date and time stamp” functionality. |
| Chat (private) |  |  |  |  |  |
|  | Simplicity |  |  |  |  |
|  |  | The 1-on-1 chat function is “hidden” in the admin menu.  None of the 5 end users was able to navigate to the chat section with ease.  Expert(s): “*A basic functionality like chatting should not be put away in the admin menu.*” | Heuristic evaluation: 1/3 experts  Think aloud: 5/5 end users | 4 | Make the private-chat accessible through a dashboard page. |
| Chat (group) |  |  |  |  |  |
|  | Simplicity |  |  |  |  |
|  |  | Deleting a person from the chat is not intuitive.  When the participant tried to delete a person from the group, the group title was clicked, hoping to find an admin menu similar to WhatsApp. It was not immediately clear the admin page had to be used. | Heuristic evaluation: 0/3 experts  Think aloud: 1/5 end users | N/A | Let the nurse remove clients from the chat group by clicking on the title bar just like in other chat apps. |
|  | Naturalness |  |  |  |  |
|  |  | It is not clear how to navigate to the group chat section.  3 participants tried to access the group chat via the admin panel, the same way as they have learned to navigate to the private chat. This was unsuccessful. | Heuristic evaluation: 0/3 experts  Think aloud: 3/5 end users | N/A | Let users navigate to the group chat the same way as they navigate to the private chat (preferably through a general chat functionality, which is not hidden in the admin page). |
|  |  | The placing of the messages is not natural. All messages are placed on the left side of the screen, which does not correspond with other messaging clients. | Heuristic evaluation: 2/3 experts  Think aloud: 0/5 end users | 2 | Place the users own messages on the right, and the other’s messages on the left of the screen. |
|  |  | The functionality to give a “heart” (or like) to a person’s message is not clear by just looking at it.  It is not clear that the heart is a clickable button, but could also be part of the text message. | Heuristic evaluation: 1/3 experts  Think aloud: 2/5 end users | 2 | The button should have a different placement on the screen, so it does not “blend in” with the message itself. |
|  | Consistency |  |  |  |  |
|  |  | There is an inconsistency in representing the number of hearts a message has received between the two interfaces.  In the client app, this is represented by a heart and the number. While in the nurse app, this is represented by a heart, an “x” and the number. | Heuristic evaluation: 1/3 experts  Think aloud: 0/5 end users | 1 | Pick one method of presenting information, and use these across both interfaces. |
| Tips |  |  |  |  |  |
|  | Simplicity |  |  |  |  |
|  |  | The app is not clear in what the nurse’s purpose with tips is.  Expert(s):*”Why does the nurse get to see the tips, just like the clients do? She is a nurse and should know these tips.”* | Heuristic evaluation: 2/3 experts  Think aloud: 0/5 end users | 3 | Give a nurse the option to add a “tip” for her clients and let her review the “tips” her clients upload. |
|  | Naturalness |  |  |  |  |
|  |  | The icon of the “tips” section was not clear to the participants.  Two participants thought it was a “help” or “info about the app” button. | Heuristic evaluation: 0/3 experts  Think aloud: 2/5 end users | N/A | Add the text “tips” to the navigation button, and use a different icon, which does not interfere with end users expectations of a general information icon. |
|  | Forgiveness and Feedback |  |  |  |  |
|  |  | Tips entries cannot be deleted or edited when a mistake is made. | Heuristic evaluation: 3/3 experts  Think aloud: 0/5 end users | 3 | Provide the option to delete and edit tips entries. |
| Admin |  |  |  |  |  |
|  | Simplicity |  |  |  |  |
|  |  | It is not intuitive to add a client to your group.  Expert(s): *“You can add a name or phone number. Does the app look in your contact list? Does it look into a list of registered Kindle users? Why can I choose between these two options?”* | Heuristic evaluation: 1/3 experts  Think aloud: 0/5 end users | 3 | Inform the user how clients are found and added. |
|  |  | The admin page feels a bit cluttered compared to the simple style of the chat and info page. | Heuristic evaluation: 2/3 experts  Think aloud: 0/5 end users | 2.5 | The admin page could be further divided into subpages.  In the current version, all the clients have their 5 icons next to them. Instead, you could only show the client names, and when clicked, show the different options in a new screen. |
|  | Naturalness |  |  |  |  |
|  |  | There are many icons on the admin page. Most of them are unclear.  Expert(s): “The heart-icon is unclear to me; the single-person-icon is unclear to me and the group-icon is unclear what it does.” | Heuristic evaluation: 2/3 experts  Think aloud: 0/5 end users | 4 | Better icons could be used (materials.io), however putting the navigation buttons in a different context could improve understandability as well  It is unnatural to access basic functionalities through a “settings” icon. Look for other possibilities of presenting important information to the nurse. E.g. via a dashboard/home screen. |
|  |  | The placing of the settings/admin button in the navigation bar does not feel natural sitting in the middle of the screen. | Heuristic evaluation: 2/3 experts  Think aloud: 0/5 end users | 2 | Settings buttons are often placed on the top/lower right of the screen. |
|  | Consistency |  |  |  |  |
|  |  | The functionality of the buttons are not consistent with the way they are portrayed.  The heart, single chat and group chat buttons are grouped close together with the same look/feel, which make the user expect the same type of result. However, the functionalities are different.  The heart and single chat buttons bring the user to another page, while the group-chat button acts like a toggle for a function, which is unknown to me. | Heuristic evaluation: 1/3 experts  Think aloud: 0/5 end users | 2 | Buttons, which navigate the user to another page, should be differently placed and/or styled from toggle buttons, which should be different from mutation buttons like delete/block. |
|  |  | The group chat toggle in the settings menu has the same icon as the group chat icon in the navigation bar. However, the buttons have different functions. | Heuristic evaluation: 1/3 experts  Think aloud: 0/5 end users | 2 | Create different icons for different functions. |
|  | Forgiveness and feedback |  |  |  |  |
|  |  | There is no way to change the group name. | Heuristic evaluation: 3/3 experts  Think aloud: 0/5 end users | 2.67 | Add an “edit” button for the nurse to change the group name. |
|  |  | The prompts “Are you sure that you want to block/remove this person?” do not include the actual person which is about to be blocked/deleted in the prompt itself. This could lead to unintended actions. | Heuristic evaluation: 2/3 experts  Think aloud: 0/5 end users | 2 | Include the person which is about to be blocked/deleted in the prompt. |
|  | Effective use of language |  |  |  |  |
|  |  | The word “Group name” could be deleted in the group overview in the grey bar for a cleaner result. | Heuristic evaluation: 2/3 experts  Think aloud: 0/5 end users | 1 | See issue. |
|  | Effective information presentation |  |  |  |  |
|  |  | The page, which welcomes the user to the admin page, and prompts to create a group (steps 1 and 2), is very cramped, and is not making effective use of the remaining screen real estate. | Heuristic evaluation: 2/3 experts  Think aloud: 0/5 end users | 2 | There is a lot of free space left to work with so the information can be presented more effective by spreading the content. |
|  | Preservation of context |  |  |  |  |
|  |  | The pop-up to block/delete a contact is off-centered and could be missed. | Heuristic evaluation: 2/3 experts  Think aloud: 0/5 end users | 1 | Show the popup in the center of the screen to grab the attention. |
|  | Minimize cognitive overload |  |  |  |  |
|  |  | There is no help or explanation on how to pick a group name. | Heuristic evaluation: 1/3 experts  Think aloud: 0/5 end users | 2 | Give a suggestion on picking a group name. |
| Profile |  |  |  |  |  |
|  | Effective information presentation |  |  |  |  |
|  |  | The green checkmark (apply) button and the red cross (cancel) button are very close to each other, and off-centered. | Heuristic evaluation: 1/3 experts  Think aloud: 0/5 end users | 1 | Centre the buttons and create more space between them. |

^a^ = rated by experts on scale 0 (no usability problem) to 4 (usability catastrophe)

N/A = not applicable

Table S2. Client interface usability evaluation results.

| Functionality | Heuristic principle | Usability problem | Detection | Severity^a^ | Recommendation |
| --- | --- | --- | --- | --- | --- |
|  |  |  |  |  |  |
| General |  |  |  |  |  |
|  | Simplicity |  |  |  |  |
|  |  | It is confusing to use multiple icons in order to show in which menu the user finds itself.  Heuristic Evaluation expert(s): *“There is a highlighted icon in the navigation bar of the corresponding page, and this same icon (which is non-clickable, but looks clickable) is also put in the right upper corner of the screen.”* | Heuristic evaluation: 2/3 experts  Think aloud: 0/5 end users | 1.5 | Only use the highlighted icon in the navigation bar, and (if necessary) use a title bar with the name of the menu on top. |
|  | Naturalness |  |  |  |  |
|  |  | Heuristic Evaluation expert(s): *“Most icons are very unintuitive”.* | Heuristic evaluation: 2/3 experts  Think aloud: 0/5 end users | 3.5 | Replace the icons by icons people know from their own mobile devices and apps. Use material.io for guidelines. |
|  |  | The red cross-icon and green check-icon have no clear meaning in their respective context. | Heuristic evaluation: 2/3 experts  Think aloud: 0/5 end users | 3.5 | These should be replaced by explicit commands like “Proceed“, “Go back”, “Save”, “Delete”, etcetera. |
|  | Consistency |  |  |  |  |
|  |  | The placement of the main icons of each page are not consistent. The single- and group chat icons are in the top left of the page; while the diary, “personal goals” and “tips” icons are on the top right. | Heuristic evaluation: 2/3 experts  Think aloud: 0/5 end users | 1 | Be consistent in icon placement on the screen. Either choose all icons to be placed right, or left. |
|  | Efficient interactions |  |  |  |  |
|  |  | Dismissing the keyboard is only possible by clicking “return”. Clicking on the screen does not work, although this works in all other apps. | Heuristic evaluation: 0/3 experts  Think aloud: 2/5 end users | N/A | Let the user dismiss the keyboard by pressing on a spot on the screen, which is not the keyboard. |
| Personal goals |  |  |  |  |  |
|  | Simplicity |  |  |  |  |
|  |  | The numbers above the golden heart-icons are unclear.  Experts and end users do not understand the context of these numbers, and how these are assigned. | Heuristic evaluation: 3/3 experts  Think aloud: 1/5 end users | 3 | Use tiny info buttons to explain the context of these numbers, or let the user watch a one-time tutorial when first launching the app. |
|  |  | It is not clear what the exact function of the “personal goals” page is, once a category is selected. | Heuristic evaluation: 2/3 experts  Think aloud: 0/5 end users | 3 | Use tiny info buttons to explain the context of these numbers, or let the user watch a one-time tutorial when first launching the app. |
|  | Naturalness |  |  |  |  |
|  |  | The pencil-icon to edit the “personal goals” seems redundant. Editing is also possible by clicking on the input field. | Heuristic evaluation: 1/3 experts  Think aloud: 0/5 end users | 3 | Since a personal goal should not be edited frequently (if not, at all), remove the option to edit by clicking on the input field. |
|  |  | The trash-icon to delete a “personal goal” seems redundant. Deleting is also possible by clicking on the input field, and erasing the text. | Heuristic evaluation: 2/3 experts  Think aloud: 0/5 end users | 2 | Since a personal goal should not be deleted frequently (if not, at all), remove the option to delete by clicking on the input field. |
|  |  | It is not clear that the “Go to completed personal goals” text is actually a clickable button. | Heuristic evaluation: 0/3 experts  Think aloud: 4/5 end users | N/A | Let the button stand out more. Use the guidelines by material.io. |
|  | Effective use of language |  |  |  |  |
|  |  | It is not clear that “completed personal goals” list the personal goals, which are already completed. | Heuristic evaluation: 0/3 experts  Think aloud: 2/5 end users | N/A | It could be that the word “completed” is hard to understand for end users. If that is the case, a replacement like “achieved” could be used.  Another cause for the problem could be that, the way that the “current personal goals” is presented, is too similar of that of “completed personal goals”, and therefore end users do not notice the difference between the pages. If this is the case, a different layout could be used to emphasize the difference between the pages. |
|  |  | The term “personal goals” is unknown to experts. | Heuristic evaluation: 2/3 experts  Think aloud: 0/5 end users | 3.5 | If the term is indeed unknown for the end users, an information button could be used for explanation. Else, this issue can be ignored. |
|  | Effective information presentation |  |  |  |  |
|  |  | The description input field might be too small for some personal goals. | Heuristic evaluation: 1/3 experts  Think aloud: 0/5 end users | 1 | Make the description field bigger to allow for more characters. |
|  |  | The green checkmark (apply) button and the red cross (cancel) button are very close to each other, and off-centered. | Heuristic evaluation: 1/3 experts  Think aloud: 0/5 end users | 1 | Centre the buttons and create more space between them. |
| Chat (general) |  |  |  |  |  |
|  | Simplicity |  |  |  |  |
|  |  | It is unclear when messages were sent. | Heuristic evaluation: 1/3 experts  Think aloud: 0/5 end users | 1 | Include “date and time stamp” functionality. |
|  | Naturalness |  |  |  |  |
|  |  | It is unknown what the heart-icon does next to the input field. | Heuristic evaluation: 2/3 experts  Think aloud: 0/5 end users | 1.5 | The intended functionality is unknown since the button does not function. Therefore a possible recommendation cannot be given. |
|  | Efficient interactions |  |  |  |  |
|  |  | There is no search functionality in chats.  Heuristic Evaluation expert(s):*”Users should be able to search for keywords in the chat for efficient information retrieval”.* | Heuristic evaluation: 1/3 experts  Think aloud: 0/5 end users | 2 | Include a search functionality in the chat. |
| Chat (private with nurse) |  |  |  |  |  |
|  | Consistency |  |  |  |  |
|  |  | It is unclear if the private chat is actually is a private conversation to one person.  Heuristic Evaluation expert(s): “*The private chat functionality gives me the illusion that it is private, but in the example I see the name “Anneke van Dam” as well as “Jolanda Seegers” sending me a message in one chat, making it a group chat.”* | Heuristic evaluation: 1/3 experts  Think aloud: 0/5 end users | 2 | Make the private chat indeed “private”. |
| Chat (group) |  |  |  |  |  |
|  | Simplicity |  |  |  |  |
|  |  | The functionality to give a heart (or “like”) to a received message is not clear by just looking at it. It is not clear that the heart is a clickable button. | Heuristic evaluation: 1/3 experts  Think aloud: 2/5 end users | 2 | The button should have a different placement on the screen, so the button does not “blend in” with the message itself. |
|  | Naturalness |  |  |  |  |
|  |  | The chat-icon in the menu is not intuitive and should be more explicit. | Heuristic evaluation: 2/3 experts  Think aloud: 1/5 end users | 2 | Use material.io for guidelines. |
|  |  | The group chat and private chat being separate functionalities is confusing.   Heuristic Evaluation expert(s)*:” I would expect the group chat to be part of the normal chat functionality. Not a separate menu button.”* | Heuristic evaluation: 2/3 experts  Think aloud: 0/5 end users | 3 | Create a chat functionality with private and group chatting as sub-functionalities. Just like how other chat apps operate (e.g. WhatsApp, Telegram, and Signal). |
|  |  | It is not clear which messages the user sends, and which are received. | Heuristic evaluation: 2/3 experts  Think aloud: 1/5 end users | 1.5 | Display the user’s messages outlined right at the screen, and others’ messages outlined left. |
|  | Consistency | The group chat does not have the heart-icon next to the input field, unlike the private chat. | Heuristic evaluation: 2/3 experts  Think aloud: 0/5 end users | 2 | Be consistent in the user-interface across both chat functionalities. (Unless the heart-input is a specific feature which only should exist in the private chat). |
| Tips |  |  |  |  |  |
|  | Naturalness | It is not clear what the plus sign is supposed to mean. | Heuristic evaluation: 0/3 experts  Think aloud: 1/5 end users | N/A | Use text (e.g. “Add”) instead of a plus sign. |
|  | Efficient interactions | There is no option to browse through existing “tips” entries. | Heuristic evaluation: 1/3 experts  Think aloud: 0/5 end users | 2 | Create an overview of tips entries where the user can scroll through. |
| Diary |  |  |  |  |  |
|  | Simplicity |  |  |  |  |
|  |  | Usage of the lock buttons next to the diary entries is unclear.  End users had problems deleting a diary post, not knowing that this lock button had to be clicked.  Heuristic Evaluation expert(s):*”The ‘lock’ buttons next to the diary posts serve no clear purpose.”* | Heuristic evaluation: 2/3 experts  Think aloud: 4/5 end users | 1 | Remove the lock buttons. |
|  | Naturalness |  |  |  |  |
|  |  | It is not clear that the lock icon in the navigation bar is supposed to present a diary.  Heuristic Evaluation expert(s):*”The lock icon seems more like a functionality to lock the main app behind a password, instead of a diary functionality.”* | Heuristic evaluation: 2/3 experts  Think aloud: 1/5 end users | 3 | Use an icon that resembles a diary, and add the title name in the navigation bar as well. (For the sake of consistency, all the navigation icons should have added titles). Check material.io for more detailed guidelines. |
|  | Consistency |  |  |  |  |
|  |  | The lock-icon which represents the diary in the navigation bar is the same icon as the lock/unlock icon in the top right corner of the diary page. The icons have different meanings, and are therefore confusing. | Heuristic evaluation: 1/3 experts  Think aloud: 0/5 end users | 1 | Use different icons for the navigation button, and the icon that shows whether the diary is locked/unlocked. |
|  | Forgiveness and feedback |  |  |  |  |
|  |  | It is not possible to change your pin code after you have created one. | Heuristic evaluation: 2/3 experts  Think aloud: 0/5 end users | 3 | Create a “my profile” menu where you can change your pin code. |
|  | Effective use of language |  |  |  |  |
|  |  | In the diary app the word “sleutel (translation: key) code” is used instead of “pin code”. This could be confusing. | Heuristic evaluation: 1/3 experts  Think aloud: 0/5 end users | 2 | Use common terminology that users will definitely understand. “pin code” is used across multiple other services that individuals use, and is therefore preferred over “sleutelcode”. |
|  | Efficient interactions |  |  |  |  |
|  |  | The pin code functionality is broken.  Heuristic Evaluation expert(s): *“The pin code has to be created again and again every time the diary is opened.”* | Heuristic evaluation: 1/3 experts  Think aloud: 0/5 end users | 4 | After creating the pin code, ask the user for the pin code instead of creating it again. |
|  | Effective information presentation |  |  |  |  |
|  |  | Edit and delete icons are too small and too close to each other. | Heuristic evaluation: 1/3 experts  Think aloud: 0/5 end users | 1 | Put them further away from each other to avoid mistakes. |
|  | Minimize cognitive overload |  |  |  |  |
|  |  | Users are unable to give a “subject” (or label) to their diary entries in order to find specific entries more easily. | Heuristic evaluation: 1/3 experts  Think aloud: 0/5 end users | 2 | Create a subject field that is presented in the overview page of diary entries. |
| Profile |  |  |  |  |  |
|  | Simplicity |  |  |  |  |
|  |  | The checkbox “VoorZorg Client” has no clear goal when making a profile. | Heuristic evaluation: 2/3 experts  Think aloud: 0/5 end users | 2 | Entirely remove the checkbox, since it has no utility. |
|  |  | The user is forced to upload a picture when creating a profile. This should be optional, since it is not necessary for the app to function properly. | Heuristic evaluation: 1/3 experts  Think aloud: 0/5 end users | 2 | Give the user the option to skip this process. |
|  | Naturalness |  |  |  |  |
|  |  | It is unclear when the profile creation is (almost) finished. The green checkmark and red cross icon have no clear meaning. | Heuristic evaluation: 2/3 experts  Think aloud: 0/5 end users | 3 | Use buttons like “next”, “back” and “create”, to explicitly let the user know what the result will be. |
|  | Forgiveness and feedback |  |  |  |  |
|  |  | Once a picture is uploaded, there is no way to change it. | Heuristic evaluation: 3/3 experts  Think aloud: 0/5 end users | 3 | Create a “my profile” menu where you can change your profile picture. |
|  |  | It is not possible to change your name after its creation. | Heuristic evaluation: 2/3 experts  Think aloud: 0/5 end users | 3 | Create a “my profile” menu where you can change your name. |
|  |  | The app gives no feedback how many steps there are for completing a Kindle profile.  Heuristic Evaluation expert(s): *“I have no idea when I am (almost) done creating my profile”.* | Heuristic evaluation: 1/3 experts  Think aloud: 0/5 end users | 2 | Give feedback about the profile creation process. How many steps are needed? When is the user almost done creating the profile? |
|  | Effective use of language |  |  |  |  |
|  |  | There is no explanation for creating a profile.  Heuristic Evaluation expert(s):*” Why should I create a profile for this app. Why it is beneficial? Who can find me? How is my privacy secured?”* | Heuristic evaluation: 2/3 experts  Think aloud: 0/5 end users | 2 | Explain why the end user needs to create a profile. |
|  |  | “Personalize your app here” is confusing when needing to upload a picture. | Heuristic evaluation: 1/3 experts  Think aloud: 0/5 end users | 2 | It should explicitly say what you want the user to do. In this case: “upload a picture”. |
| N/A |  |  |  |  |  |
|  | Preservation of context |  |  |  |  |
|  |  | N/A | N/A | N/A | N/A |

^a^ = rated by experts on scale 0 (no usability problem) to 4 (usability catastrophe)

N/A = not applicable
